# Supplementary figures and images for: A multi-sensor array for detecting and analyzing nocturnal avian migration
Source: PeerJ. 2023 Aug 30;11:e15622. doi: 10.7717/peerj.15622 (PMC10474833; doi:10.7717/peerj.15622)

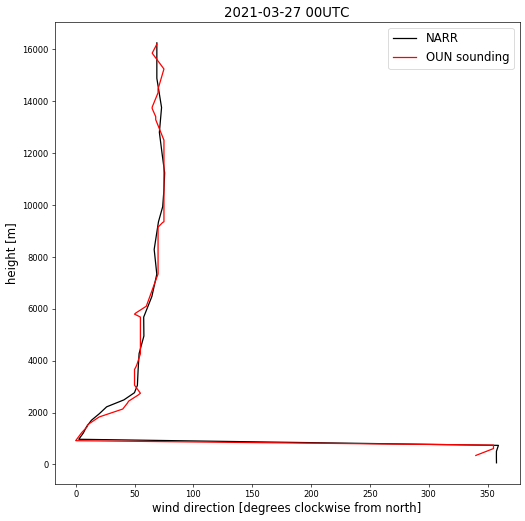

Supplement: Supplemental Information 3 — A wind profile was created for each three-hour interval between 0 and 12 UTC on 3/26/2021, 3/27/2021, 3/28/2021, 3/29/2021, 4/24/2021, and 5/25/2021. [file peerj-11-15622-s003.zip › wind_profiles/validate_direction.png]

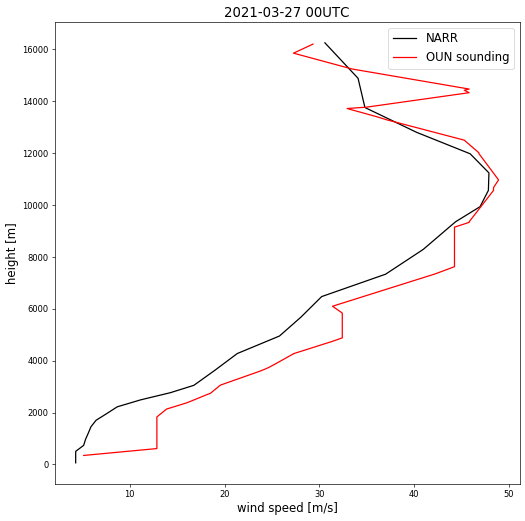

Supplement: Supplemental Information 3 — A wind profile was created for each three-hour interval between 0 and 12 UTC on 3/26/2021, 3/27/2021, 3/28/2021, 3/29/2021, 4/24/2021, and 5/25/2021. [file peerj-11-15622-s003.zip › wind_profiles/validate_speed.png]
